# Development of an Empirically Calibrated Model of Esophageal Squamous Cell Carcinoma in High-Risk Regions

**DOI:** 10.1155/2019/2741598

**Published:** 2019-05-22

**Authors:** Zhenhua Wang, Qiang Zhang, Bin Wu

**Affiliations:** ^1^Division of Gastroenterology and Hepatology, Key Laboratory of Gastroenterology and Hepatology, Ministry of Health, State Key Laboratory for Oncogenes and Related Genes, Renji Hospital, School of Medicine, Shanghai Jiao Tong University, Shanghai Institute of Digestive Disease, China; ^2^Department of Oncology, Putuo Hospital, Shanghai University of Traditional Chinese Medicine, China; ^3^Medical Decision and Economic Group, Department of Pharmacy, Renji Hospital, South Campus, School of Medicine, Shanghai Jiaotong University, China

## Abstract

**Objective:**

This study constructs, calibrates, and verifies a mathematical simulation model designed to project the natural history of ESCC and is intended to serve as a platform for testing the benefits and cost-effectiveness of primary and secondary ESCC prevention alternatives.

**Methods:**

The mathematical model illustrates the natural history of ESCC as a sequence of transitions among health states, including the primary health states (e.g., normal mucosa, precancerous lesions, and undetected and detected cancer). Using established calibration approaches, the parameter sets related to progression rates between health states were optimized to lead the model outputs to match the observed data (specifically, the prevalence of precancerous lesions and incidence of ESCC from the published literature in Chinese high-risk regions). As illustrative examples of clinical and policy application, the calibrated and validated model retrospectively simulate the potential benefit of two reported ESCC screening programs.

**Results:**

Nearly 1,000 good-fitting parameter sets were identified from 1,000,000 simulated sets. Model outcomes had sufficient calibration fit to the calibration targets. Additionally, the verification analyses showed reasonable external consistency between the model-predicted effectiveness of ESCC screening and the reported data from clinical trials.

**Conclusions:**

This parameterized mathematical model offers a tool for future research investigating benefits, costs, and cost-effectiveness related to ESCC prevention and treatment.

## 1. Introduction

The global disease burden contributed by esophageal cancer is approximately 10 million disability-adjusted life-years (DALYs) [1], which is ranked 11^th^ worldwide and 6^th^ in China for all neoplasms [2, 3]. In esophageal cancers, 90% of the annual 456,000 incident cases were esophageal squamous cell carcinoma (ESCC). The risk factors for ESCC are multifactorial and strongly population-dependent, such as cigarette smoking and heavy alcohol consumption in the Western population and high-temperature foods, diet, oral health, and the microbiome in the Eastern population [4]. Due to the late stage at diagnosis for most patients and limited treatment options, the case fatality rate of ESCC is high, and the prognosis is poor [5]. Thus, identification of primary or secondary prevention strategies to reduce the disease burden of ESCC is a public health priority. From the perspective of the high-risk regions of ESCC, such as China, where approximately 40% of the world's DALY related to esophageal cancers occur, implementation of the ESCC screening program might offer a feasible option to reduce the disease burden.

To explore feasible strategies in Chinese high-risk regions, several ESCC screening studies based on endoscopic examinations with mucosal iodine staining and index biopsy have been performed for several decades [6–8] and showed that early detection and subsequent treatment could notably reduce the cumulative incidence of ESCC versus the control group (4.17% vs. 5.92%, respectively; P < .001) and cumulative mortality (3.35% vs. 5.05%, respectively; P < .001). To measure the public health benefits of an ESCC screening program, several factors must be considered: the potential natural history of ESCC, the heterogeneity of risk conferred by age and region, the effectiveness of treating precancerous lesions in interfering with the route to cancer, and the feasibility of implementing a secondary prevention program at the population level. All of these potential factors and examination of all possible alternatives in all populations cannot be considered in one clinical study. Therefore, a mathematical simulation model in a decision-analytic framework designed to project the natural history of ESCC is valuable because it synthesizes the best available biological, epidemiologic, and economic data. This model can aid in decision-making by assessing the appropriateness, effectiveness, and cost-effectiveness of different strategies and finding the most sensitive factors.

The aim of this study is to present the development of an ESCC policy model, including a comprehensive framework of the model structure with the best available clinical and epidemiological inputs, calibration techniques and endpoints, and model validation. By identifying and using a series of good-fitting parameter sets, this validated model can serve as a foundation and platform for future evaluations that supplies evidence for prevention and management of ESCC with the goal of improved patient outcomes and optimized resource utilization.

## 2. Methods

### 2.1. Overview

This mathematical model illustrates the natural history of ESCC with the goal of constructing a universal platform for further health economic and policy research. We depict the methodological processes of model construction, including the definition of the model structure or health states, identification of essential model inputs and assumptions, and measurement of parameters by calibrating the model to empirical targets. The main empirical data sources for model inputs were taken from the published literature. The calibration approach identifies a series of good-fitting unknown parameters sets that could generate model outputs that are consistent with descriptive observed data [13]. Using this empirically calibrated model, we can assess the potential impact of ESCC interventions on the outcomes.

### 2.2. Model Structure and Assumptions

**Figure 1:**
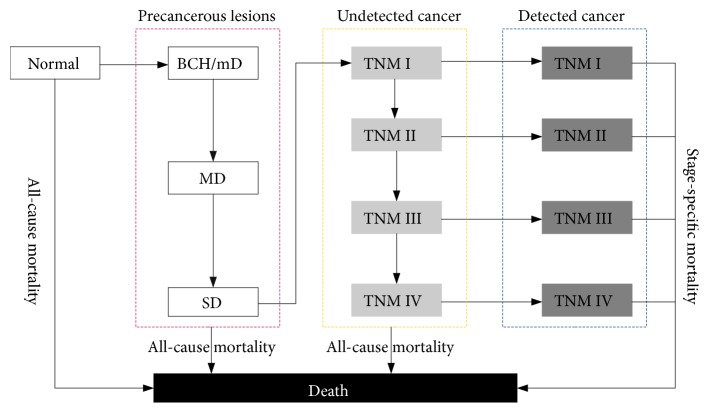
Schematic of the model structure. BCH, basal cell hyperplasia; mD, mild dysplasia; MD, moderate dysplasia; SD, severe dysplasia.

We constructed an ESCC policy model simulation model of ESCC with 13 health states, including normal, basal cell hyperplasia (BCH)/mild dysplasia (mD), moderate dysplasia (MD), severe dysplasia (SD), undetected cancer in TNM stages I, II, III, and IV, detected cancer in TNM stages I, II, III and IV, and death, as shown in Figure 1. Because the risks of BCH are highly similar to that of mD and the morphological distinction between BCH and mild dysplasia can be difficult [9], BCH and mD were treated as the first early precancerous lesions. The ESCC policy model applies the Markov state transition technique and is programmed in R [14]. The model simulates the natural history of ESCC among the population followed from age 15 to age 100 or death (whichever comes first). At the start of a simulation, the hypothetical population was assumed to be in the normal health state. In the states of normal and precancerous lesions, age-dependent all-cause mortality probabilities based on the life tables of the World Health Organization (WHO) member states (2011) were used in the analysis [10]. Based on expert opinion, the model assumed that undetected cancer and detected cancer in TNM stage I also incurs natural all-cause mortality. However, once cancer is developed, cancer-specific mortality rates are affected.

Based on the current evidence [5], the model could not allow for direct progression from the normal health state to the cancer state. In accordance with the consensus of an expert panel on the high risk of recurrent precancerous lesions, no regression among health states was assumed, although regression among the health states related to precancerous lesions might be possible. Additionally, other health states such as acanthosis and esophagitis were not considered. These assumptions could lead to a parsimonious model, which allowed us to simplify the model development and calibration processes.

### 2.3. Parameterization

To establish the parameter values in the model, a comprehensive literature search was conducted to identify prospective cohort studies related to the transition probabilities of ESCC. We calibrate the probability of developing precancerous lesions (BCH/mD), progression of precancerous lesions and the progression rate of cancer, and clinical detection dependent on the TNM stages (Table 1). Transitions between the preclinical precancerous lesions states and from preclinical to clinical ESCC are assumed to be independent of age, except for the parameters related to the rate of developing BCH/mD from the normal state, which is written as follows:

(1) log⁡λ1=β0+∑k=13δAk≤aget<Ak+1·agetβk+∑j=3kAjβj−1−βj

- *β*_*0*_ is the baseline log-risk;
- *δ(·)* is an indicator function with* δ(x)* = 1 when x is true and* δ(x)* = 0 otherwise;
- *age(t)* is the population age at time t;
- A_1_ = 15, A_2_ = 40, A_3_ = ∞ (effectively 100 years old);
- *β*_*1*_ and *β*_*2*_ is the log-risk based on age (age group: A_1_-A_2_ and A_2_-A_3_).

The risk of developing MD from BCH/mD was negatively dependent on the duration of BCH/mD, which is written as shown:

(2) $$\begin{array}{c} \log\left(\;\lambda_{2}\;\right)=\alpha_{0}-\alpha_{1}T_{d} \end{array}$$

- *α*_*0*_ is the baseline log-risk;
- T_d_ is the duration in the BCH/mD health state;
- *α*_*1*_ is the log-risk of the duration time.

The incidence rate of cancer in the SD health state was derived from a 13-year cohort study in the Chinese population [9]. We estimated the age-specific prevalence of precancerous lesions between the ages of 40 and 69 years and the incidence of ESCC by extrapolating cross-sectional trend data on individuals between the ages of 30 and 85+ years. Due to uncertainty related to the estimates within each study and heterogeneity among different cohorts based on the study design and bias, the upper and lower boundaries of each parameter were defined to be widely inclusive of (1) the confidence intervals reported by studies (when available), (2) the upper and lower boundaries reported from different data sources, and (3) expert opinion.

### 2.4. Empirical Calibration

Due to a paucity of reports in this area, few transition data were found in the publications. Thus, we use calibration to identify the parameters sets of transition probabilities to maintain the consistence between the model outputs and calibration targets. Calibration targets were extracted from published data and include the age-specific prevalence of precancerous lesions (Table 2), incidence of ESCC cancer in three Chinese high-risk regions (Yanting, Cixian, and Lin counties) from 2003 to 2007, and the proportions of ESCC TNM stage at diagnosis from a Chinese cohort study [11, 12], which defined a total of 34 targets (18 precancerous, 12 ESCC, and 4 proportions of ESCC TNM stage): the proportion of TNM stages of newly diagnosed ESCC [11, 15]; prevalence of precancerous lesion in high-risk areas [16]; incidence and mortality rates of esophageal cancer in Cixian [17]. We specified likelihood functions for each target, assuming each follows an independent binomial distribution. The chi-squared goodness-of-fit (GOF) function, a goodness-of-fit score equal to -2 × the sum of the log-likelihood scores for all of the calibration targets, was used as the metric to measure the model outputs for all of the calibration targets. We defined the best-fitting parameter sets as those with the lowest GOF scores.

We calibrated the model based on the genetic algorithm, which is a heuristic search and optimization technique inspired by natural evolution in which the fittest parameter set dominates over the weaker ones [18]. In this process, a group of model parameter sets (analogous to parent chromosomes) were randomly created from the parameter space defined by the plausible ranges of the model parameters (as described above), which were used to measure the GOF score of each parameter set by running the simulation model. The proportional difference between the GOF score of a parameter set and the largest GOF score among all tested parameter sets was used as the probability of selecting the parameter set for creation of one or more offspring (analogous to children chromosomes), where the crossover operator (recombination between every selected two parents chromosomes) was applied to form the next-generation chromosomes, and random changes of values of individual parameters (analogous to gene mutation) were performed on these offspring chromosomes [19]. In this model calibration, the algorithm was run with 80% crossover between pairs of chromosomes, 25% mutation in a parent chromosome, and 100,000 iterations.

To refer to the uncertainty of the estimated parameter value, a Bayesian method was used to generate a distribution of the calibrated parameters by applying the technique of sampling importance resampling [20]. In this process, the best-fitting parameter set was used as the point estimate for generating an initial 1,000,000 samples and a resample. Using the likelihood values of each parameter set as sampling weights, resampling from the original parameter sample with replacement was performed to obtain the unique weighted sample size, which is a useful metric for understanding the distribution and upper and lower boundaries of each parameter [20].

### 2.5. Hypothetical Screening Analysis: Mimic Example of Future Model Application

Screening for ESCC is currently recommended in the Chinese high-risk regions. The recent endoscopic screening for esophageal cancer in a Chinese trial was the first population-based randomized controlled trial to evaluate the outcomes of endoscopy screening for ESCC. This trial enrolled a total of 33,948 individuals from 668 villages in rural Hua County, including 15,188 in the screening arm, which received screening endoscopy with pathologic diagnosis [7]. Because the rural incidence rate of esophageal cancer in the age range of 45-69 years was estimated as 184.07/100,000 in Hua County and 392.80/100,000 in the other three Chinese high-risk regions (Yanting, Cixian, and Lin counties), the risk ratio of approximately 0.469 could be applied to adjust the risk of developing BCH/mD in Hua County. As an application of our model used to assess total esophageal high-grade lesions, we applied the model to assess the number of precancerous lesions and the number of cancers that could be detected if screening were implemented.

Another Chinese study enrolled 45,386 participants in an EC high-incidence region for evaluation of whether an endoscopic screening and intervention program could reduce mortality caused by ESCC in Cixian County [8], where the hazard ratio of ESCC is approximately 1.30 compared with the average rate in three Chinese high-risk regions (Yanting, Cixian, and Lin county). In the screening arm, nearly 48.6% of patients were screened once by endoscopy with Lugol's iodine staining, and those with dysplasia or occult cancer were treated. This 10-year follow-up study reported the hazard ratio of endoscopic screening versus no screening in the cumulative incidence of ESCC in the age 40-69 population. The sensitivity and specificity of endoscopic detection with mucosal iodine staining for identification of high-grade (moderate and severe) squamous dysplasia or ESCC were 96% and 63%, respectively [6, 21].

## 3. Results

### 3.1. Model Fit

**Figure 2:**
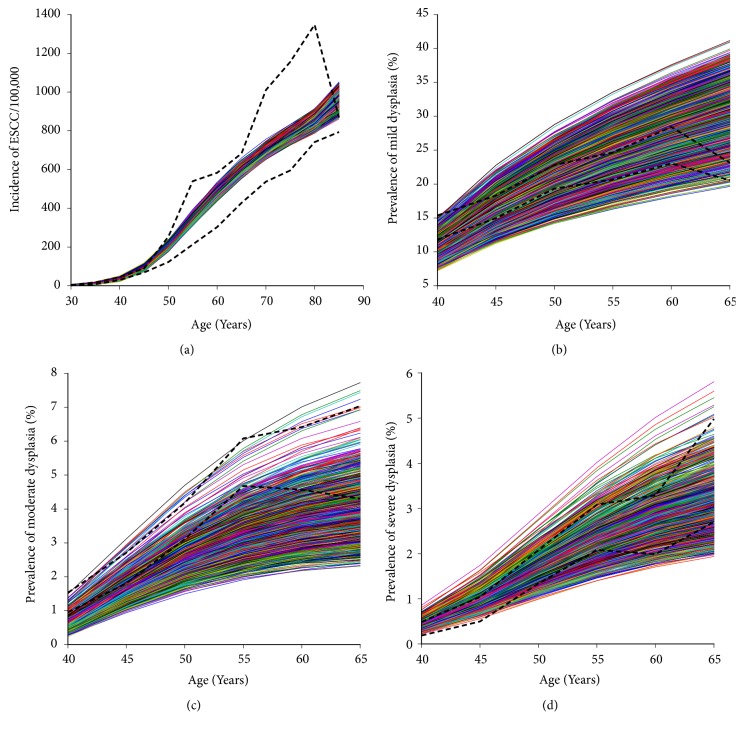
Model fit to calibration targets on (a) ESCC incidence, (b) mD prevalence, (c) MD prevalence, and (d) SD prevalence using good-fitting parameter sets in the Chinese high-risk region. Bold black dashed lines, lower and upper boundaries of observed age-specific epidemiological data; nonbold color lines, model output for 952 selected good-fitting parameter sets. ESCC, esophageal squamous cell carcinoma; mD, mild dysplasia; MD, moderate dysplasia; SD, severe dysplasia.

The model was fit to our calibration targets using 952 good-fitting parameter sets, precancerous lesions prevalences, and ESCC incidence stratified by specific age groups. The fits of the ESCC incidence rates (Figure 2(a)) are nearly covered by the ranges of the observed data. The fits for mild, moderate, and sever dysplasia prevalence showed the largest variation (Figures 2(b)–2(d)), which covered the lower and upper limits of the observed data. The incidence in the 20-29-age group is not displayed in Figure 2(a) due to poor resolution in the y-axis, but the fit was similar or better compared with the other age groups presented.

**Figure 3:**
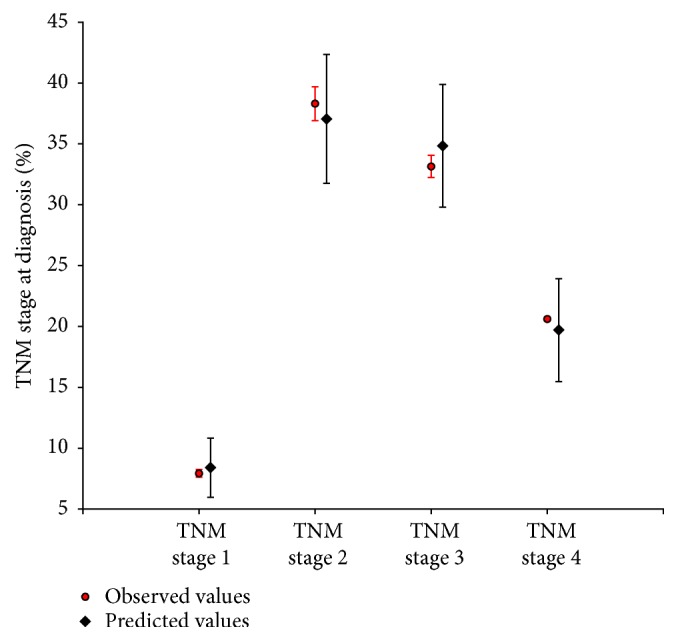
Model fit to calibration targets on TNM stage data using good-fitting parameter sets.

Figure 3 shows the estimates of TNM stage at diagnosis, which were good because the model predictions are in agreement with the observed data.

### 3.2. Model Verification

As an initial test and verification of the model, we simulated the effects of screening the entire 45-69-year-old population in Hua County with iodine staining endoscopy (Table 3). Our model predicted an underlying prevalence of any esophageal high-grade lesions at 0.99% (95% CI: 0.72-1.28%) for the age 45-69 population with 0.59% (95% CI: 0.34-0.85%) for severe dysplasia (precancerous lesions) and 0.4% (95% CI: 0.29-0.52%) for ESCC (undetected cancer), which were consistent with the reported data [7]. However, the predicted prevalence of BCH/mD was lower than that of the reported data, and the predicted prevalence of MD was higher than that of the reported data.

The verification results in Cixian County showed that the predicted 10-year incidences of ESCC in the control and intervention arms were lower than the reported values. However, the hazard ratio was well matched for the predicted and reported data (Table 3).

## 4. Discussion

Motivated by a need to inform policy decision makers of potential secondary prevention programs for ESCC, we incorporated the available evidence to construct a model of ESCC and empirically calibrated this model to data in the Chinese high-risk regions. This approach found multiple good-fitting parameter sets that fit comparably well with the reported epidemiologic data. These parameters gained the lowest GOF scores with a global manner that generated the predicted values which are not uniformly higher or lower than the observed values in different TNM stages in Figure 3. By performing verification analyses of endoscopic screening for ESCC using the good-fitting parameter sets, the model creates an illustration of the uncertainty in policy outcomes caused by uncertainty in the model parameters. As such, in our verification example, although the policy model predicted that the truncated prevalence of SD and ESCC in the age 45-69 population might be matched with the reported data, the model also indicated that estimates of the truncated prevalence of BCH/mD and MD deviate substantially if we explicitly contemplate the potential uncertainty related to disease progression. The potential reason for the deviations from the predicted and reported data and the difference of verification results between the Hua and Cixian County study is that the reported prevalence and incidence values were derived from mixed populations with varied risk factors, and our predicted data were estimated from a hypothetical birth cohort, which did not track the impact of known and suspected risk factors because the etiology of ESCC is multifactorial and strongly population-dependent [4].

We presented details of the methodological process used in the construction of the model, with the aim of transparency to give assurances of model integrity and validity [22]. The fundamental strength of the ESCC policy model is the comprehensive and strict approach we applied to construct and validate it with predefined calibration targets, calibration using a well-known automated parameter search algorithm, and quantitative evaluation of model output fit to calibration targets. It is additional model strength that the model included three precancerous lesions states and four undetected ESCC states, which is vital to inform the comparative effectiveness of early intervention in the real world. The previous policy model for ESCC did not consider undetected cancer and the impact of age on the incidence [23]. The model might measure and evaluate the underlying disease burden associated with ESCC that was revealed by cross-sectional ESCC screening [24]. This observation is crucial to the next comparative effectiveness study on weighing the costs and potential outcomes of secondary prevention in epidemic regions.

In addition to estimating early detection and treatment, the ESCC model offers a foundation on which to test which clinical interventions are more appropriate for treating patients. After surgery for ESCC, 9-50% of patients are expected to experience cancer recurrence [25, 26]. By simulation of the hypothetical cohort and objective evaluation of tailored approaches used to select high-risk individuals for endoscopy [27], the use of novel diagnostics and screening techniques [28], adjuvant targeted immunotherapy [29, 30], and risk-stratification algorithms to optimize surveillance for tumor development and progression can be made more cost-effective [31].

We chose epidemiological data from high-risk regions as the primary calibration target because these are the highest quality data available in China. A vital component in modeling ESCC is the consideration of all precancerous lesions and undetected cancer, given the collocation and difficulty in definitive early diagnosis of malignancy. The health and financial implications of all precancerous lesions and undetected cancer must be considered. In an attempt to track the natural history of ESCC, it is imperative that we include growth of both precancerous lesions and undetected cancer. However, observed data related to disease progression and regression among precancerous lesions are limited, and for this reason, future long-term cohort studies are necessary [31].

Our calibration techniques contain weaknesses. First, the development and validation of a health policy model is in the early stage of health care decision research. No consensus exists on model construction or how “good” the model must fit to ensure full illustration of disease natural history, and thus even a rigorously determined “good-fitting parameter set” is subject to analytical selections. Currently, the growing consensus is that a systematic and reproducible approach for model calibration should be recommended [32]. In this analysis, a systematic likelihood-based approach using GOF scores for quantitative evaluation was applied for model calibration and validation and was widely used in development of other cancers, such as gastric cancer, cervical cancer, and colorectal cancer [33–35]. Although other approaches such as *χ*2 and the Bayesian approach were also used in other diseases [20, 36], we did not compare the differences in performance of these approaches. Second, the model did not allow regression among the health states of precancerous lesions because of a paucity of evidence from a small sample size addressing this issue [37] and the simplification of model development and validation. Third, the calibrated parameters of the model are cohort averages and do not mirror deviations among individuals who might produce differences due to different risk factors, such as high-temperature foods, diet, oral health, cigarette use, and heavy alcohol consumption [4], which require further research. The approach used in this ESCC policy model mirrored the tradeoffs and balance of data availability, computer capacity, convenience of implementation, and our relatively modest aim of producing widely qualitative insight for decision-making.

In summary, we illustrated the results of the construction and validation of a health policy model for ESCC. In future adjustment, additional data such as economic and health preferences related to esophageal cancer could be integrated into the model for future studies. The model can also be updated to incorporate new evidence as it becomes available in the future. Our ultimate aim for the ESCC model is for it to serve as a platform for future health policy and service studies that produce evidence to improve the health outcomes and optimize resource consumption associated with esophageal cancer.

## Figures and Tables

**Table 1:** Model input parameters.

Parameter	Expected value (SD)	Source
Progression		
Normal → BCH/mD	*β*_0_ = -8.96 (0.461)	Derived from calibration
	*β*_1_ = 0.20481 (0.01815)	
	*β*_2_ = 0.00002 (0)	
	*β*_3_ = 0.00836 (0.00134)	
BCH/mD → MD	*α*_0_ = -3.359 (0.171)	Derived from calibration
	*α*_1_ = 0.03668 (0.0095)	
MD → SD	15.38%	Derived from calibration
SD → Undetected ESCC (TNM I)	20.94%	[9]
Undetected ESCC (TNM I) → Undetected ESCC (TNM II)	15.38%	Derived from calibration
Undetected ESCC (TNM II) → Undetected ESCC (TNM III)	20.94%	Derived from calibration
Undetected ESCC (TNM III) → Undetected ESCC (TNM IV)	46.65%	Derived from calibration
Detection rate		
Undetected ESCC (TNM I) → Detected ESCC (TNM I)	54.87%	Derived from calibration
Undetected ESCC (TNM II) → Detected ESCC (TNM II)	32.31%	Derived from calibration
Undetected ESCC (TNM III) → Detected ESCC (TNM III)	3.86%	Derived from calibration
Undetected ESCC (TNM IV) → Detected ESCC (TNM IV)	34.32%	Derived from calibration
Mortality		
All-cause mortality	Chinese life-table	[10]
Disease-specific mortality (TNM I)	Chinese life-table*∗*	[10]
Disease-specific mortality (TNM II)	15.15%	[11, 12]
Disease-specific mortality (TNM III)	35.97%	[11, 12]
Disease-specific mortality (TNM IV)	56.47%	[11, 12]

*∗*It was assumed to be equivalent with normal population after complete resection.

ESCC, esophageal squamous cell carcinoma; BCH, basal cell hyperplasia; mD, mild dysplasia; MD, moderate dysplasia; SD, severe dysplasia.

**Table 2:** Calibration targets.

Age Group (year)	ESCC incidence (per 100,000)	mD prevalence (%)	MD prevalence (%)	SD prevalence (%)
30-34	3.4	-	-	-
35-39	12.4	-		-
40-44	42.4	13.1	1.24	0.34
45-49	93.1	16.7	2.26	0.77
50-54	238.7	21.1	3.62	1.71
55-59	475.8	22.6	5.37	2.58
60-64	519.6	25.8	5.5	2.64
65-69	636.8	24.3*∗*	5.67*∗*	3.84*∗*
70-74	878.9	-	-	-
75-79	925.1	-	-	-
80-84	1145.1	-	-	-
≥85	967.6	-	-	-

*∗*≥65 years old.

ESCC, esophageal squamous cell carcinoma; mD, mild dysplasia; MD, moderate dysplasia; SD, severe dysplasia.

**Table 3:** Verification results.

	Observed values	Predicted values
Hua County study (aged 45–69 years)		
Prevalence of BCH/mD (%)	22.04	8.56 (95% CI: 6.48 - 10.64)
Prevalence of MD (%)	0.57	1.03 (95% CI: 0.72 - 1.34)
Prevalence of SD (%)	0.41	0.59 (95% CI: 0.4 - 0.79)
Prevalence of ESCC (%)	0.33	0.4 (95% CI: 0.31 - 0.5)
Prevalence of total oesophageal high-grade lesions (%)*∗*	0.74	1 (95% CI: 0.78 - 1.21)
Cixian County study (40–69 years)		
10-year incidence of ESCC in control arm (%)	5.92	3.54
10-year incidence of ESCC in screeing arm (%)	4.17	2.47
HR (screening VS. control)	0.698	0.694

*∗*Total number of severe squamous dysplasia, squamous carcinoma in situ, and squamous cell carcinoma.

ESCC, esophageal squamous cell carcinoma; BCH, basal cell hyperplasia; mD, mild dysplasia; MD, moderate dysplasia; SD, severe dysplasia; HR, hazard ratio.

## Data Availability

No additional data are available.

## Additional Points

*Provenance and Peer Review. *This study was not commissioned; it was externally peer reviewed.
